# Lived Challenges Contributing to Mental Illness Relapse and Coping Strategies Used by Teachers in Limpopo Province

**DOI:** 10.3390/ijerph22071048

**Published:** 2025-06-30

**Authors:** Thembi Nkomo, Mokoko Percy Kekana, Mabitsela Hezekiel Mphasha

**Affiliations:** Department of Public Health, University of Limpopo, R71 Tzaneen Road and University Street, Polokwane 0727, South Africa

**Keywords:** social determinants of health, mental illness relapse, vulnerability, environmental factors

## Abstract

Mental illness relapse among teachers presents a growing public health concern, particularly in under-resourced settings, where social and structural factors often go unaddressed. This study aimed to explore challenges outside the workplace that contribute to mental illness relapse among public school teachers in Limpopo Province and how they cope with them. Guided by the Stress-Vulnerability Model, a qualitative explorative phenomenological design was employed. Fourteen participants with a documented history of existing mental illness and mental illness relapse were purposively selected across four different hospitals. The data were collected through in-depth, face-to-face semi-structured interviews until data saturation was reached. The interviews were audio-recorded, transcribed verbatim, and analysed using Tesch’s open coding method. The findings revealed unstable home environments, community-level stigma, inadequate institutional support, and systemic barriers to mental healthcare access. Moreover, the participants rely on family members for support and on spiritual practices to cope, highlighting gaps in formal support systems. Addressing these overlooked challenges is critical to reducing relapse resulting from social and systematic challenges, promoting mental health equity, and sustaining teacher resilience in underserved communities. This study calls for collaborative efforts from policymakers, educational institutions, healthcare providers, and community leaders, including faith-based organisations, to develop integrated mental health strategies. Such strategies can promote mental health equity, reduce stigma, and support sustainable teacher well-being in vulnerable communities.

## 1. Introduction

Mental illness comprises various clinically diagnosable conditions, such as major depressive disorder, schizophrenia, and bipolar disorder. Mental illness remains a leading contributor to the global burden of disease and the foremost cause of disability worldwide, often resulting in significant functional impairment, reduced quality of life [1], and adverse impacts on individuals’ health and social well-being [2]. Teachers, as frontline professionals in the education system, are increasingly experiencing mental health challenges, with many reporting episodes of mental illness relapse [3]. A growing body of research points to high levels of stress and challenges among teachers as a significant concern. While stress prevalence among teachers is widely documented, Agyapong et al. [4] revealed the variability in teacher stress worldwide, with prevalence rates ranging from 8.3% (moderate) to 87.1% (severe). In Africa, stress rates among teachers are similarly high at 95.9% in Morocco [5], 72% in Nigeria [6], and 81.2% in South Africa [7]. Various studies have found that teachers are consistently exposed to a unique set of challenges that make them particularly susceptible to stress, including high workloads, administrative burdens, inadequate support, and constant performance pressures [8,9]. Unlike many other professionals, teachers often perform emotional labour in environments that lack essential psychosocial and institutional safeguards [10]. These challenges are especially pronounced for teachers already living with a diagnosed mental illness, where such stressors may act as potent triggers for mental illness relapse. While this body of research provides valuable insight into the extent of stress among teachers, less is known about the challenges they face outside the work environment, many of which relate to the social determinants of health, such as social aspects in their communities, the accessibility and quality of mental health services, and the dynamics of their home environments. These factors contribute to stress and increase the risk of mental illness relapse, as they are not merely background variables but powerful drivers of health outcomes [11]. Thus, a critical gap persists in understanding how the intersection of social and systemic factors affects teachers with existing mental illness, how they contribute to mental illness relapse in this high-risk group, and how teachers cope with these challenges in their daily lives. This study aimed to address this gap by exploring challenges outside the workplace that contribute to mental illness relapse among teachers and how they cope with them. The central research question guiding this study was as follows: What challenges outside the workplace contribute to mental illness relapse among teachers, and how do they cope with these challenges? By understanding their challenges and coping mechanisms, this study sought to generate insights that can guide locally responsive support strategies for teachers with mental illness in South Africa, while also contributing valuable insights to global conversations on how social factors outside the workplace shape mental health outcomes and mental illness relapse risk. This research forms part of an ongoing broader doctoral project aimed at developing and validating a strategy to minimise mental illness relapse among public school teachers in Limpopo province, South Africa. Ultimately, this study advocates for a shift away from individualised, clinical-only models of mental health toward integrated, socially responsive frameworks that prioritise both structural reform and the voices of teachers themselves.

### Theoretical Framework

In order to understand how challenges outside the workplace affect teachers with existing mental illness, how they contribute to mental illness relapse, and how they cope with the challenges, we used the Stress-Vulnerability Model developed by Zubin and Spring [12]. This model suggests that individuals have different levels of vulnerability to mental illness, and excessive stressors can trigger or exacerbate these vulnerabilities. The model highlights three essential factors:

Biological vulnerability—this refers to a person’s inherited or existing mental illness, such as a history of depression, bipolar disorder, or schizophrenia.

Stress—this refers to everyday pressures or difficult life events that can overwhelm a person, such as financial strain, family conflict, or lack of community support.

Protective factors—this refers to the support and skills that help reduce the impact of stress, including strong social networks, healthy coping strategies, and access to mental health services.

This model not only framed our understanding of how challenges outside the workplace affect teachers with existing mental illness, how they contribute to mental illness relapse, and how they cope with the challenges. The model has also guided the identification of key intervention points to inform the development of a context-specific, preventative strategy and policies that go beyond clinical care.

To ensure theoretical alignment, the model directly informed the study’s qualitative phenomenological design, which aimed to capture the lived experiences of teachers outside their workplace. It also shaped the development of the semi-structured interview guide, which included open-ended questions designed to probe the types of challenges (stress) participants encountered and the coping strategies (protective factors) that they used. This alignment between theory, design, and data collection ensured that the study not only captured the depth but also remained anchored in a framework capable of informing real-world, preventative strategies for reducing mental illness relapse among teachers.

## 2. Materials and Methods

### 2.1. Study Design

For this empirical study, a qualitative phenomenological design was chosen as the most appropriate methodology to meaningfully explore the lived experiences of teachers with existing mental illness and battling mental illness relapse. This approach centres on capturing participants’ experiences as they are consciously perceived, deliberately minimising preconceptions or researcher biases to ensure authentic representation [13]. By prioritising the subjective realities of teachers, the phenomenological design facilitated an in-depth investigation of how challenges outside the workplace affect teachers with existing mental illness, how they contribute to mental illness relapse, and how they cope with the challenges within the geographic context of Limpopo [14]. This methodological choice enhanced the applicability of the findings by providing insights that reflect the real-world complexities teachers face, thereby informing contextually relevant interventions. Ultimately, the approach yielded a rich, comprehensive understanding of the significance and meaning these lived experiences hold, which is critical for developing effective, culturally sensitive mental health support strategies [15].

### 2.2. Study Setting and Participants

This study was conducted at four different hospitals in Limpopo Province, which serve as key centres providing care and rehabilitation to patients with mental illness from various communities and districts across the province. Conducting the study in hospital settings rather than schools was to ensure purposive sampling of the participants, who were engaged in ongoing care, making them suitable for understanding the phenomenon under investigation. The selected facilities included one regional hospital, one tertiary academic hospital, and two specialised psychiatric hospitals (one state and one private). Patients were recruited from both hospital wards and outpatient departments. The inclusion of multiple institutions was deliberate, aiming to capture a broad and diverse range of mental health service contexts. This approach ensured representation from general regional care to specialised psychiatric treatment, enabling richer and more comprehensive data on the mental health challenges experienced by public school teachers. Including both public and private psychiatric hospitals also allowed for the examination of potential differences in access to care, treatment modalities, and institutional support, which are critical for understanding challenges influencing teacher well-being. Furthermore, recruiting across several hospitals facilitated capturing the geographic and socio-economic diversity of Limpopo’s rural, semi-urban, and peri-urban communities. A non-probability purposive sampling method was employed to enhance the validity and reproducibility of the results by selecting public school teachers diagnosed with mental illness who had experienced multiple relapses [16]. Eligible participants were required to be stable and, if admitted, to be awaiting discharge, to ensure their willingness and capacity to communicate their experiences articulately and reflectively [17]. Those who were unstable or declined consent were excluded. Stability and capacity to participate were first assessed by psychiatrists and nursing staff, who then referred eligible teachers to the primary investigator. In total, 14 participants with documented mental illness and a history of mental illness relapse more than once were sampled. Focusing on participants with multiple relapses allowed for a richer understanding of how ongoing social, personal, and systemic stressors contribute to relapse trajectories, which aligns with the aims of this phenomenological inquiry and the guiding Stress-Vulnerability Model.

The type of mental illness was not a criterion. The study focused on teachers with any type of documented mental illness and who had experienced a mental illness relapse more than once, regardless of diagnosis, to better understand the role of stressors and support systems in relapse. This aligns with the Stress-Vulnerability Model [12], which emphasises that relapse can occur in any mental illness when stress exceeds an individual’s coping capacity, regardless of the specific diagnosis.

### 2.3. Data Collection

Ethical approval was first obtained from the university, followed by permissions from the Limpopo Province Department of Health, which acts as the gatekeeper for state hospitals, and from the institutional review boards of the participating hospitals, including a regional hospital, a tertiary academic hospital, and a specialised psychiatric hospital. Data were also collected at a private specialised psychiatric hospital, where admitting doctors provided verbal consent for patient interviews. A pilot study involving two participants at a regional hospital was conducted to test the interview guide for clarity and relevance; these participants were not included in the main study, and no modifications to the guide were required [18,19]. The interview guide was developed based on the Stress-Vulnerability Model [12] to frame questions around the participants’ experiences of mental illness relapse and social stressors. All participants provided written informed consent after receiving detailed information about the study and voluntarily participated without incentives. Data were collected by the primary investigator through semi-structured, face-to-face interviews using open-ended questions, all audio-recorded. All the interviews were conducted by the primary investigator, who is fluent in English, Sepedi, and Xitsonga. The primary investigator’s linguistic fluency significantly contributed to establishing rapport with all the participants, and this created a comfortable and culturally sensitive environment that encouraged the participants to speak openly about their challenges and coping strategies outside their workplace, which has the potential to trigger their mental illness relapse. Probing and clarification questions were employed to elicit depth and richness in the participants’ responses. Triangulation was further enhanced through direct observation, detailed field notes, and reflective dialogue with the participants [20]. While the interviews were primarily conducted in English, the participants occasionally expressed themselves in Sepedi or Xitsonga, with translations provided where necessary.

The primary investigator’s role extended beyond data collection to include reflexive awareness of their positionality and potential influence on the research process. Conscious efforts were made to mitigate researcher bias by maintaining a reflective journal throughout the data collection and analysis, continuously questioning assumptions and interpretations. This reflexivity helped to enhance the trustworthiness and credibility of the findings by ensuring that the participants’ voices were authentically represented. Potential participants were initially assessed by nurses and psychiatrists, who referred eligible teachers—those with a history of multiple mental illness relapses, who were clinically stable, and if admitted, were awaiting discharge—to the primary investigator. Those deemed unstable or who declined participation were excluded. Data collection took place over two weeks, with the interviews lasting approximately 45 minutes each. A total of 14 participants were interviewed, with sampling ceasing upon reaching data saturation, defined as the point at which no new themes or information emerged [21]. The leading interview question posed to all participants was as follows: “What challenges outside the workplace contribute to your mental illness relapse, and how do you cope with these challenges?” This leading question was supplemented by sub-questions and probes to elicit rich, detailed descriptions of the participants’ lived experiences regarding the social determinants of health and their coping strategies [22]. Data collection continued until saturation was reached, with sufficient richness and depth of information obtained to fully capture the complexity and nuance of the teachers’ lived experiences.

### 2.4. Data Analysis

The data were analysed manually following Tesch’s eight-step method of qualitative data analysis, as outlined by Creswell [23]. The process began with Step 1, which involved listening to the audio recordings, transcribing the interviews verbatim, and translating portions from Xitsonga and Sepedi into English. The transcripts were read repeatedly to gain familiarity with the data, and relevant field notes were integrated into the corresponding transcripts. In Step 2, two copies of each transcript were prepared: a master copy and another for noting initial impressions and reflections by the researcher. One transcript was selected for detailed preliminary analysis, during which the researcher noted underlying meanings and impressions in the margins, numbered the paragraphs, and identified key ideas.

Step 3 involved carefully examining each transcript to identify and cluster related topics into columns, using colour coding and abbreviations to develop preliminary codes. These initial codes were not final but were revisited and refined continuously as further data were analysed. In Step 4, codes were systematically assigned next to relevant text segments and examined for emerging categories. These categories were provisional and subject to modification in response to ongoing analysis. Step 5 entailed identifying the most descriptive terms for topics and grouping related topics into broader categories, while also exploring the relationships between them. During Step 6, categories were further refined, defined clearly, and alphabetised, acknowledging that this was part of an iterative analytical process. Step 7 involved assembling all data relevant to each category for a preliminary analysis and thematic refinement. Themes were then finalised based on the depth and coherence of the data, guided by the Stress-Vulnerability framework [12]. Step 8, involving re-coding, was deemed unnecessary as no new codes emerged that required reclassification or adjustment.

To enhance trustworthiness and reduce researcher bias, intercoder reliability was ensured by involving a second independent coder, who reviewed a sample of transcripts and coding schemes. Discrepancies were discussed until consensus was reached, fostering consistency in the interpretation of the data. Additionally, the primary investigator maintained a reflexive journal throughout the analysis process to critically reflect on personal assumptions and potential biases, thereby promoting transparency and rigour in the data interpretation.

### 2.5. Ethics

Following ethical clearance from the University of Limpopo and obtaining permission to conduct the study from the Limpopo Province Department of Health and the institutional review boards of all participating hospitals prior to data collection, informed consent was obtained from all participants after a thorough explanation of the study’s purpose, procedures, potential risks, and benefits. The participants were informed that their involvement was entirely voluntary and that they could withdraw at any time without penalty. To ensure confidentiality and anonymity, all interviews were conducted in private settings within the hospital premises, away from other patients and staff. Personal identifiers were removed from the transcripts, and pseudonyms were assigned to the participants during the transcription and analysis. The audio recordings and transcripts were securely stored in password-protected files accessible only to the primary investigator and authorised personnel. The data were handled in compliance with ethical standards to prevent any potential breach of privacy. Given the sensitive nature of discussing mental health challenges, care was taken to minimise any psychological distress during the interviews. The interviewer remained attentive to participants’ comfort levels, offering breaks or discontinuation if distress was observed. Referral pathways to counselling and support services were made available should participants require further assistance.

### 2.6. Trustworthiness Measures

Credibility—Prolonged engagement with the participants and the use of triangulation through the interviews, field notes, and reflective dialogue enhanced the depth and accuracy of the findings. Member checking was conducted by sharing the summaries of the key themes with the participants to confirm accurate representation of their experiences [24].

Dependability—An audit trail documenting all phases of the data collection and analysis was maintained, including reflective journals, coding decisions, and theme development. This transparency allowed for replication and verification of the study process [25].

Confirmability—Reflexivity was emphasised, with the primary investigator maintaining a reflexive journal to monitor and mitigate personal biases throughout the data collection and analysis. The involvement of an independent second coder in reviewing the transcripts and codes further reduced the influence of individual subjectivity [26].

Transferability—Detailed descriptions of the study context, participant characteristics, and data collection processes are provided to enable readers to assess the applicability of the findings to other similar settings [24].

## 3. Results

Table 1 shows that six participants were male public school teachers, eight participants were female public school teachers, and they all had a history of mental illness relapse. The majority of the participants were mid-career teachers (aged 36–46), and they all had experienced relapses more than three times.

Table 2 indicates three themes and six subthemes that emerged from the analysed data. The themes identified in this study were theoretically informed and guided by the Stress-Vulnerability Model [12], which underpinned the study’s conceptual framework. This model emphasises the interaction between individual vulnerability, external stressors, and protective factors.

### 3.1. Theme 1: Social, Cultural, and Personal Factors Affecting Teachers’ Mental Health

This theme yielded two subthemes. It explores the various social, cultural, and personal factors that influence teachers’ mental health. It further investigates the impact of social isolation and cultural stigma, highlighting how these factors heighten feelings of loneliness and anxiety, increasing the risk of mental illness relapse.

#### 3.1.1. Subtheme 1.1: Personal Challenges and Unstable Home Environments Add to Emotional Distress

The participants reported that personal challenges such as forgetfulness, strained relationships, and family conflicts significantly contributed to their emotional distress. They reported problems with remembering important tasks such as medical appointments, and some mentioned family tensions and betrayal in relationships. The participants’ experiences are further outlined in the following quotations:

“I honestly forget my appointment dates. they write them for us on the appointment card at the hospital, and I always forget where I put it. I live with my grandmother, who is also not in a state to remind me of my hospital appointment dates.”

(Participant 4)

“I live with my late uncle’s son; that boy is troublesome… he abuses substances, and he is now stealing from me. I don’t have peace both at home and at work. I drag my feet to go home after work, and I drag my feet to go to work in the morning. Sometimes I just wonder if I will ever have peace.”

(Participant 6)

Field notes: Participant 6’s tone became heavier when mentioning her late uncle’s son, showing signs of frustration and distress. There was also a noticeable shift in her mood when describing her work routine, expressing reluctance to engage in both home and work life.

“My husband is cheating on me with one of my colleague’s friends… guess what… it is a man, he is cheating on me with a man… I’m a laughing stock in the community and at work… I even took my ring off because of the gossip at work and in my community. The worst part is he denies it, but my cousin showed me their pictures in the guy’s Facebook. I think the guy uploaded those pictures just to hurt me, but my husband denies it. He says they are just friends, but I know he is lying and this affects me because I can’t cope when I’m at work and when I’m at home.”

(Participant 8)

Field notes: Participant 8 exhibited signs of emotional distress; she frequently paused to compose herself while discussing the situation. Her body language became tense, with crossed arms, especially when mentioning the affair and the gossip, and her facial expression shifted from anger to sadness, highlighting a mix of betrayal and confusion.

“Being single does not help me either because I knock off from a lonely, unsupportive, and unbearable work environment to a lonely house with no one to talk to.”

(Participant 10)

#### 3.1.2. Subtheme 1.2: Societal Stigma and Mental Health Taboos Lead to Social Judgment and Exclusion

The participants shared how social stigma and deep-rooted taboos surrounding mental illness subjected them to judgment, exclusion, and social isolation. They described being perceived as “crazy,” being labelled, and having their condition defined not only for them but also for their families. The participants’ experiences are further outlined in the following quotes:

“In my community, mental illness is still viewed as a taboo; they look at you as if you are crazy, and no one wants to be associated with you. I really don’t have a sense of belonging in my community, and I stopped attending gatherings such as funerals and weddings because I know they will be gossiping about me.”

(Participant 1)

“Mental illness is still viewed as a taboo in my community. Even myself, I never thought I will ever be diagnosed with mental illness, I did not choose to have mental illness and that is something that the community I live in does not understand, because my children are also labelled as the children of a father with mental illness. They hardly engage in community events because of such labelling, and this also affects me.”

(Participant 9)

“The stigma on mental illness within the community and at school makes it hard for me to feel part of the team and for me to accept myself with the condition that I have.”

(Participant 13)

### 3.2. Theme 2: Barriers to Accessing Adequate Mental Healthcare

This theme yielded two subthemes. It highlights the various obstacles teachers face in accessing adequate mental healthcare, such as systematic challenges and financial constraints, and they are further explored below:

#### 3.2.1. Subtheme 2.1: Systematic Barriers to Mental Healthcare Access in Public Hospitals Discourage Treatment Seeking

The participants reported experiencing long waiting times for appointments, and some reported waiting months to see a psychologist. The participants mentioned that being visibly singled out for mental health consultations discouraged them from seeking treatment. They also reported having experienced dismissive attitudes of healthcare professionals. Their experiences are further noted in the quotes below:

“When I go consult a clinical psychologist at a government hospital, I’m given an appointment date in the next two months because they are fully booked. This means that for the next two months, I will just be in distress because there will be no help for me.”

(Participant 1)

“At the hospital where I consult, people with mental illness consult on specific days and specific consultation rooms. It then becomes obvious on my reason for consultation. The queues are always long, and the way the nurses talk to us, it’s as if we are not human beings. I get demotivated to go for my checkup because of such things.”

(Participant 5)

Field notes: Participant 5 likely experienced frustration and sadness, which was evident in the way she described her consultation experience. The use of words like “demotivated” and “as if we are not human beings” shows a sense of emotional exhaustion and a feeling of being devalued or disrespected.

“The psychiatrist referred me to the psychologist. Guess what… they gave me an appointment date that was in two months’ time, they said they were fully booked… Our government institutions are failing us, hey, and I was in dire need of psychological assistance.”

(Participant 10)

#### 3.2.2. Subtheme 2.2: Financial Constraints Compel Teachers to Rely on Government Healthcare, Resulting in Missed Consultations and Inconsistent Treatment

The participants reported financial strains of not affording private mental healthcare and also admitted to missing appointments and running out of medication due to the challenges of accessing treatment, leading to setbacks in managing their mental health. Their responses are outlined in the quotes below:

“I took out about two separate loans for my previous boyfriend, and he never paid any of them. He left me in debt, and I have not recovered, because I’m still paying for the loans. I’m not able to afford medical aid due to such financial difficulty, and I sometimes get demotivated to go and consult at our government hospitals because of the long queues, especially where we retrieve our files before going to see the psychiatrists.”

(Participant 4)

“I just want to sort out my finances so I can consult with a private psychiatrist. I end up running out of medication because I get demotivated to consult in our government hospital because of the long queues and the stigma, and this sets me back and makes me to relapse.”

(Participant 5)

Field notes: Participant 5 displayed signs of frustration and exhaustion, such as rubbing her forehead and fidgeting. She presented with a mix of hope (wanting to afford private care) and frustration (being stuck in the public system). The phrase “I just want to sort out my finances” suggests determination, but the reference to relapse shows that her setbacks are frequent, leading to discouragement. Her facial expressions shifted between moments of resolve and visible disappointment, and she presented with downcast eyes when speaking about relapsing.

“I cannot afford medical aid because of the state of my financial circumstances. I consult at a government hospital, and you know their queues are always long. This makes me lazy to go for reviews at the hospital.”

(Participant 12)

### 3.3. Theme 3: Coping Strategies in Managing Social Stressors

This theme yielded two subthemes. It explores the participants’ personal coping mechanisms and their spiritual aspects. This is further explored in the following subthemes:

#### 3.3.1. Subtheme 3.1: Supportive Family Members Serve as Protective Measures

Participants reported that they rely on their family members for psychological support. Their experiences are further quoted below:

“My sister is my biggest supporter. She’s the one who also accompanies me to my checkups. Her support strengthens me a lot. I talk to her about everything, and that’s how I cope with my struggles.”

(Participant 3)

“My uncle’s wife is my pillar. although she lives far away, I’m able to call her, and she always knows what to say.”

(Participant 6)

“Luckily, I have a supportive husband. It’s just that when I’m frustrated, I take the frustration home, and now my husband gets to pay for other people’s sins.”

(Participant 11)

#### 3.3.2. Subtheme 3.2: Spiritual Aspects Serve as a Coping Mechanism

The participants reported that they pray and that prayer gives them strength on a daily basis. Their experiences are outlined in the quotes below:

“I mostly read my bible and pray when I’m faced with challenges. I search for comforting bible scriptures and pray. It keeps me going.”

(Participant 2)

“I don’t have anyone to talk to about everything that I am going through. I find it hard to make friends and to trust people. I read my bible, listen to sermons, and pray, and that helps me to cope with my daily struggles.”

(Participant 13)

“I pray, prayer is the most important thing that keeps me in place and gives me strength to wake up and face the world again”

(Participant 14)

## 4. Discussion

The Stress-Vulnerability Model [12] guided this study to explore challenges outside the workplace that contribute to mental illness relapse among teachers and how they cope with them. When exploring the teachers’ challenges, namely the stressors as defined by the Stress-Vulnerability Model [12], it was found that the participants’ combination of family conflicts and relationship difficulties were some of the causes of their stressors, and this contributed to their feelings of helplessness and emotional exhaustion, ultimately causing mental illness relapse as they were left unattended to.

The lack of adequate support systems further intensified the participants’ distress, forcing them to navigate these overwhelming challenges without the necessary resources or assistance. Therefore, it is clear that these individuals require a comprehensive, holistic approach to address their well-being. Without this, their emotional burdens will remain unattended, leading to a continued decline in their mental health and overall functioning. Another stressor that came up was societal stigma. These findings align with existing literature on the detrimental effects of societal stigma on individuals with mental illness. Ahad et al. [27] and Subu [28] highlighted how stigma leads to delayed treatment and poor quality of life, with individuals being socially isolated and rejected. The participants’ reports align with these findings, as they experienced being avoided by community members due to their mental illness, and also experienced their children being labelled as the children of a “father” with mental illness. The stigma faced by the participants extended beyond the individual, affecting their families and social lives, further isolating them from their communities.

However, while the findings of Ahad et al. [27] and Subu [28] emphasise the negative consequences of societal stigma, the results of this study challenge the assumption that individuals with mental illness always experience psychological resilience or reactance in response to stigma, as suggested by Brehm [29] and Rush et al. [30], because the participants in this study expressed the emotional toll that stigma has on them. This directly contradicts the claims of psychological reactance, which suggest that individuals might resist negative stereotypes and develop more positive self-perceptions. Instead, the participants, as noted in this study, struggled with self-acceptance, and the emotional impact of stigma suggests that the emotional toll may be more severe than what has been typically described in resilience models. Another study also found that one of the challenges perpetuating high levels of stress among teachers is cultural stigma surrounding mental illness, which often discourages teachers from seeking help for fear of professional judgment or social exclusion [31,32].

The results also highlight the role of systemic and organisational factors in exacerbating the negative impacts of stigma. While Sayed et al. [33] focused on delayed treatment, poor medication adherence, and inconsistent follow-ups, the findings in this study go beyond the personal consequences of stigma to examine the institutional and organisational contexts in which stigma occurs. The problems in government healthcare facilities make it difficult for people to obtain the mental health support they need [34]. Long waiting times and staff shortages often leave individuals feeling frustrated and ignored. In many public hospitals, mental health patients are treated differently, and this stigma discourages them from seeking help, and this was seen in the participant’s responses, as it was also mentioned that people with mental illness consult on a specific day and in specific consultation rooms. Endale et al. [35] found that the lack of mental health professionals is a major challenge, and this was clear in the participants’ experiences, as they struggled to get proper care, specifically consultations with clinical psychologists. Moreover, the South African health system dedicates only 5% of its national health budget to mental health, most of which is allocated to urban psychiatric hospitals—leaving rural educators with minimal formal support [36,37].

Another serious issue is the lack of privacy in public hospitals, which made the participants feel uncomfortable about attending appointments. Even though mental health is included in healthcare policies, there is a big gap between what is promised and what actually happens. Without real improvements, such as better funding, more trained staff, and stronger policies, public mental health services will continue to fail those who need them most. Moreover, the participants’ experiences highlight how financial struggles severely impact their ability to manage mental health conditions effectively. Ryu and Fan [38] found that financial stress is closely linked to increased psychological distress, which aligns with the accounts of the participants who cannot afford private mental healthcare. As a result, they are forced to rely on underfunded and overstretched government facilities, where long waiting times, inadequate staffing, and stigma further discourage consistent treatment [27]. When looking at the protective factors, the participants’ experiences highlight the significant role that supportive family members and spiritual aspects play in assisting them to cope on a daily basis. These findings are also located in a study conducted in Limpopo Province by Nkomo and Kekana [39] on caregivers of persons with mental illness. It was found that caregivers played significant roles in supporting their loved ones with mental illness, such as accompanying them for their medical checkups and providing emotional support. This then insinuates that the participants rely on external assistance to cope with their stressors emanating from the social determinants of health. Although these coping mechanisms offer emotional relief, they are not sufficient in addressing the systemic issues contributing to mental health distress. The over-reliance on such external support mechanisms may hinder the participants from seeking professional care or engaging fully with treatment options. This suggests an urgent need for more inclusive and supportive communities, access to mental health services in state hospitals, such as psychological assistance, and policies that go beyond clinical care.

While the teachers interviewed had varying personal circumstances: seven were married and seven were single; eight were female and six were male—there were shared experiences across the group. Most participants were diagnosed with major depressive disorder, and common challenges such as community stigma and personal challenges emerged throughout. From the perspective of the Stress-Vulnerability Model [12], the differing levels of personal support acted as protective or risk factors in managing their mental illness. While all participants were exposed to similar stressors, such as community stigma and personal challenges, their capacity to cope appeared to be moderated by the strength and nature of their social support networks. Married teachers with supportive partners potentially had a buffer against stress-related relapse, while single teachers’ reliance on extended family showed variability in the stability and accessibility of support. Thus, while living conditions and status differed, the interaction between individual vulnerability, environmental stressors, and available support systems provides insight into the participants’ shared and divergent experiences with mental illness.

### Limitations

Although the sample size of 14 participants was determined by data saturation, it remains a limitation of the study. While the study offers valuable insights into teachers’ lived experiences of non-workplace-related challenges contributing to mental illness relapse and the coping strategies they employ, the relatively small and context-specific sample may limit the transferability of the findings to other regions of South Africa or different international contexts. Future research with larger, more diverse samples across varying geographic and sociocultural settings is recommended to enhance the generalizability and depth of understanding of these complex experiences.

## 5. Conclusions

By using the Stress-Vulnerability Model [12], this study highlighted the critical role of social determinants of health in contributing to mental illness relapse among public school teachers in Limpopo Province, South Africa. The available data should be used to inform the development of targeted mental health support strategies for teachers, focusing on reducing stressors and strengthening protective factors in line with the Stress-Vulnerability Model. It can guide policy recommendations, identify systemic gaps, and support advocacy for improved mental health interventions, while also serving as a foundation for future research. This study further calls upon various stakeholder collaborations with the academic community to do further research on how they can assist teachers to develop internal protective factors, upon community leaders and faith-based organisations in communities to assist in creating inclusive and supportive communities and minimising stigma in communities, and upon policymakers to develop policies that go beyond clinical care and ensure sufficient mental health staff in public healthcare settings. This will promote sustained recovery and reduce the burden of mental illness relapse in South Africa’s public education system and further minimise the contribution of social determinants of health in mental illness relapse.

## Figures and Tables

**Table 1:** Participants’ demographic information.

Participant Number	Age	Sex	Diagnosis	Number of Relapses	Marital Status
1	38	Female	Bipolar disorder	5	Single
2	26	Female	Major depressive disorder	3	Single
3	46	Female	Major depressive disorder	4	Married
4	35	Female	Major depressive disorder	3	Single
5	37	Female	Bipolar disorder	4	Single
6	50	Female	Schizophrenia	6	Single
7	38	Male	Major depressive disorder	4	Married
8	45	Female	Bipolar disorder	5	Married
9	40	Male	Schizophrenia	4	Married
10	39	Male	Major depressive disorder	6	Single
11	42	Female	Major depressive disorder	5	Married
12	36	Male	Generalised Anxiety Disorder	3	Married
13	50	Male	Schizophrenia	4	Single
14	53	Male	Bipolar disorder	6	Married

**Table 2:** Themes and subthemes.

Themes	Subthemes
Social, Cultural, and Personal Factors Affecting Teachers’ Mental Health.	1.1: Personal challenges and unstable home environments add to emotional distress.
	1.2: Societal stigma and mental health taboos lead to social judgment and exclusion.
Barriers to Accessing Adequate Mental Healthcare.	2.1: Systematic barriers to mental healthcare access in public hospitals discourage treatment seeking.
	2.2: Financial constraints compel teachers to rely on government healthcare, resulting in missed consultations and inconsistent treatment.
Coping Strategies in Managing Social Stressors.	3.1: Supportive family members serve as protective measures.
	3.2: Spiritual aspects serve as a coping mechanism.

## Data Availability

The original contributions presented in this study are included in the article. Further inquiries can be directed to the corresponding author.
